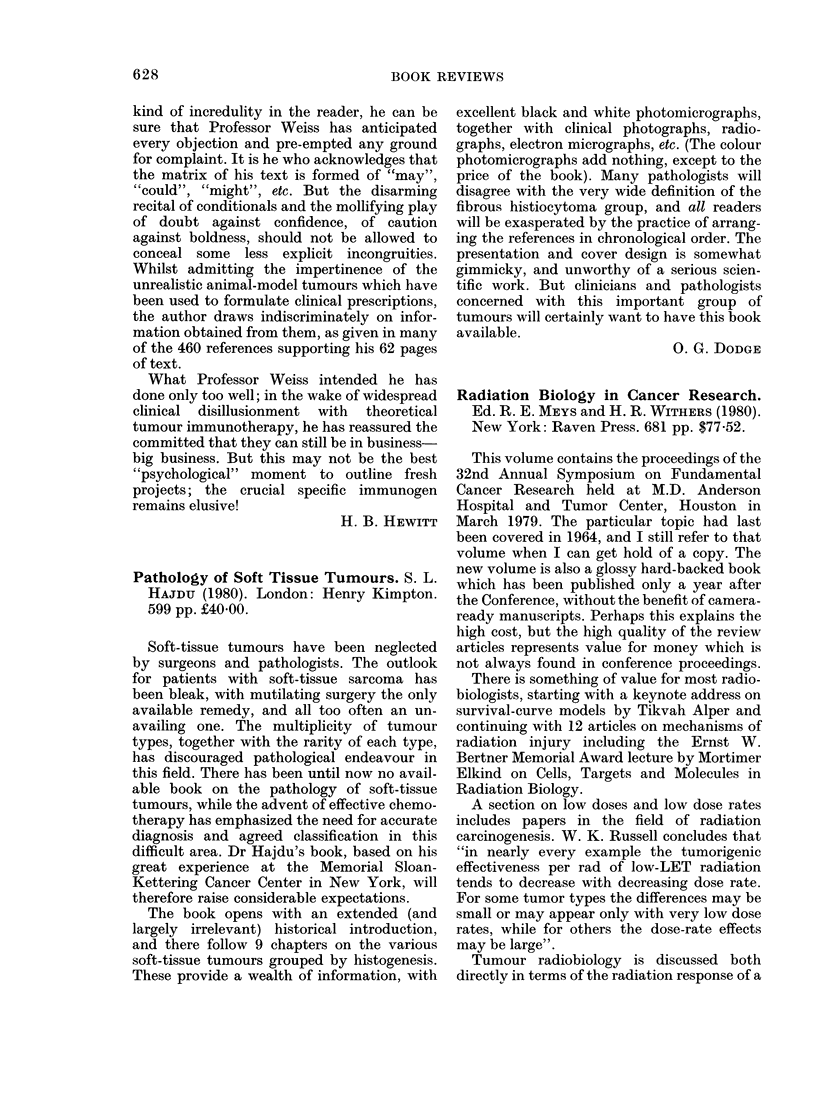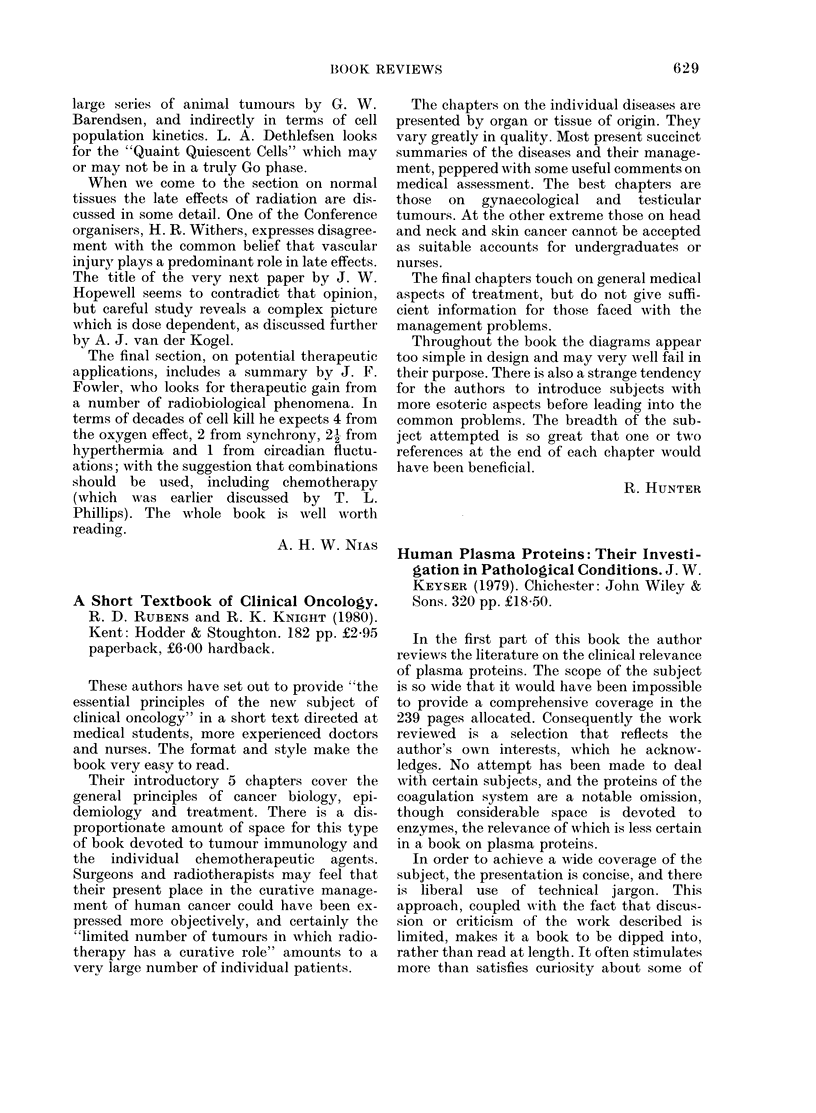# Radiation Biology in Cancer Research

**Published:** 1980-10

**Authors:** A. H. W. Nias


**Radiation Biology in Cancer Research.** Ed. R. E. MEYS and H. R. WITHERS (1980). New York: Raven Press. 681 pp. $77·52.

This volume contains the proceedings of the 32nd Annual Symposium on Fundamental Cancer Research held at M.D. Anderson Hospital and Tumor Center, Houston in March 1979. The particular topic had last been covered in 1964, and I still refer to that volume when I can get hold of a copy. The new volume is also a glossy hard-backed book which has been published only a year after the Conference, without the benefit of camera-ready manuscripts. Perhaps this explains the high cost, but the high quality of the review articles represents value for money which is not always found in conference proceedings.

There is something of value for most radiobiologists, starting with a keynote address on survival-curve models by Tikvah Alper and continuing with 12 articles on mechanisms of radiation injury including the Ernst W. Bertner Memorial Award lecture by Mortimer Elkind on Cells, Targets and Molecules in Radiation Biology.

A section on low doses and low dose rates includes papers in the field of radiation carcinogenesis. W. K. Russell concludes that "in nearly every example the tumorigenic effectiveness per rad of low-LET radiation tends to decrease with decreasing dose rate. For some tumor types the differences may be small or may appear only with very low dose rates, while for others the dose-rate effects may be large".

Tumour radiobiology is discussed both directly in terms of the radiation response of a large series of animal tumours by G. W. Barendsen, and indirectly in terms of cell population kinetics. L. A. Dethlefsen looks for the "Quaint Quiescent Cells" which may or may not be in a truly Go phase.

When we come to the section on normal tissues the late effects of radiation are discussed in some detail. One of the Conference organisers, H. R. Withers, expresses disagreement with the common belief that vascular injury plays a predominant role in late effects. The title of the very next paper by J. W. Hopewell seems to contradict that opinion, but careful study reveals a complex picture which is dose dependent, as discussed further by A. J. van der Kogel.

The final section, on potential therapeutic applications, includes a summary by J. F. Fowler, who looks for therapeutic gain from a number of radiobiological phenomena. In terms of decades of cell kill he expects 4 from the oxygen effect, 2 from synchrony, $2\frac{1}{2}$ from hyperthermia and 1 from circadian fluctuations; with the suggestion that combinations should be used, including chemotherapy (which was earlier discussed by T. L. Phillips). The whole book is well worth reading.

A. H. W. NIAS